# Regional thermal hyperemia in the human leg: Evidence of the importance of thermosensitive mechanisms in the control of the peripheral circulation

**DOI:** 10.14814/phy2.14953

**Published:** 2021-08-04

**Authors:** Nuno Koch Esteves, Oliver R. Gibson, Ashraf W. Khir, José González‐Alonso

**Affiliations:** ^1^ Centre for Human Performance, Exercise and Rehabilitation College of Health, Medicine and Life Sciences Brunel University London Uxbridge UK; ^2^ Division of Sport, Health and Exercise Sciences Department of Life Sciences College of Health, Medicine and Life Sciences Brunel University London Uxbridge UK; ^3^ Department of Mechanical and Aerospace Engineering College of Engineering, Design and Physical Sciences Brunel University London Uxbridge UK

**Keywords:** blood flow, heat, hemodynamics, thermal mechanisms

## Abstract

Hyperthermia is thought to increase limb blood flow through the activation of thermosensitive mechanisms within the limb vasculature, but the precise vascular locus in which hyperthermia modulates perfusion remains elusive. We tested the hypothesis that local temperature‐sensitive mechanisms alter limb hemodynamics by regulating microvascular blood flow. Temperature and oxygenation profiles and leg hemodynamics of the common (CFA), superficial (SFA) and profunda (PFA) femoral arteries, and popliteal artery (POA) of the experimental and control legs were measured in healthy participants during: (1) 3 h of whole leg heating (WLH) followed by 3 h of recovery (*n *= 9); (2) 1 h of upper leg heating (ULH) followed by 30 min of cooling and 1 h ULH bout (*n *= 8); and (3) 1 h of lower leg heating (LLH) (*n *= 8). WLH increased experimental leg temperature by 4.2 ± 1.2ºC and blood flow in CFA, SFA, PFA, and POA by ≥3‐fold, while the core temperature essentially remained stable. Upper and lower leg blood flow increased exponentially in response to leg temperature and then declined during recovery. ULH and LLH similarly increased the corresponding segmental leg temperature, blood flow, and tissue oxygenation without affecting these responses in the non‐heated leg segment, or perfusion pressure and conduit artery diameter across all vessels. Findings demonstrate that whole leg hyperthermia induces profound and sustained elevations in upper and lower limb blood flow and that segmental hyperthermia matches the regional thermal hyperemia without causing thermal or hemodynamic alterations in the non‐heated limb segment. These observations support the notion that heat‐activated thermosensitive mechanisms in microcirculation regulate limb tissue perfusion during hyperthermia.

## Graphical abstract

The present study investigated the effects of full and segmental leg heating on regional limb temperature, haemodynamics and tissue oxygenation. We found that prolonged whole leg hyperthermia induces a sustained and profound elevation in upper and lower leg blood flow, and that segmental leg hyperthermia matches the regional hyperaemia during whole leg hyperthermia, independent of changes in temperature, blood flow or tissue oxygenation of the non‐heated segment. Increased blood flow occurred without changing perfusion pressure or mean conduit artery diameter but were concurrent with elevations in local tissue oxygenation, blood velocity, flow rate, vascular conductance and shear rate, which were all positively related with the rise in local temperature. These observations support that local hyperthermia increases peripheral tissue blood flow through heat‐activated thermosensitive mechanisms in the microcirculation.

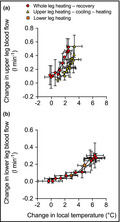

## 1 INTRODUCTION

Passive whole and segmental limb hyperthermia increase local tissue perfusion in association with elevations in calculated limb vascular conductance (Kalsi et al., 2017; Keller et al., 2010; Pearson et al., 2011; Romero et al., 2017). Nevertheless, the exact vascular locus in which hyperthermia increases blood flow and tissue perfusion within the limb vascular tree remains elusive. Classic whole body hyperthermia studies suggest that central hemodynamic factors—that is, changes in mean arterial pressure and cardiac output—modulate the regulation of peripheral blood flow during hyperthermia (Blair et al., 1960; Edholm et al., 1957). Recent evidence, however, reveals that single leg heating elicits similar leg blood flow responses to moderate whole body hyperthermia (*T*_c_ +1ºC), despite large differences in systemic hemodynamics and temperature responses (Chiesa et al., 2016; Pearson et al., 2011). During local hyperthermia, limb blood flow is elevated in a local tissue temperature‐dependent manner, suggesting that local thermosensitive mechanisms in the limb vasculature rather than central hemodynamic factors play a crucial role in limb tissue blood flow regulation during hyperthermia (Chiesa et al., 2016; Heinonen et al., 2011; Pearson et al., 2011). Nonetheless, it remains unknown whether heating a limb segment—that is, the upper or lower leg—solely increases temperature and blood flow in the heated region or whether it would also evoke responses in the adjacent non‐heated limb segment.

Conduit artery blood flow is the product of vascular conductance and perfusion pressure gradient (Laughlin, 1999). In local limb hyperthermic conditions where perfusion pressure remains stable, an increase in vascular conductance—and thus an increase in arterial diameter and/or blood velocity—could explain the hyperthermia‐induced hyperemia. Nevertheless, whether local hyperthermia increases conduit artery diameter remains equivocal, with studies reporting increases (Kalsi et al., 2017), decreases (Thomas et al., 2017), or no changes in arterial diameter (Chiesa et al., 2016; Coombs et al., 2021; Pearson et al., 2011; Teixeira et al., 2017). Moreover, hyperthermia may directly act on the conduit artery supplying blood to the heated region by increasing arterial stiffness/decreasing arterial distensibility rather than altering diameter, as seen during incremental exercise (Pomella et al., 2018). The current literature suggests that central and/or regional arterial stiffness is unchanged during acute hyperthermia (Ganio et al., 2011; Moyen et al., 2016; Schlader et al., 2019) and declines following the cessation of heating (Lee et al., 2018; Sugawara & Tomoto, 2021; Thomas et al., 2017). To our knowledge, however, no study has explored the effects of hyperthermia on arterial distensibility using local techniques—such as the *PU*‐loop or ln(*D*)*U*‐loop (Feng & Khir, 2010; Khir et al., 2001). This could provide further evidence to support the therapeutic potential of local hyperthermia for the treatment of circulatory diseases and/or rehabilitation (Brunt et al., 2016; Coombs et al., 2019; Thomas et al., 2017), as arterial stiffness—which might rise during long‐term bed rest, leg immobilization, and sedentary behavior (Bleeker et al., 2005; Bohn et al., 2017; van Duijnhoven et al., 2010; Mortensen et al., 2012)—is commonly associated with increases in cardiovascular mortality (Vlachopoulos et al., 2010).

The aim of the present study was threefold. First, to comprehensively investigate the tissue temperature and oxygenation profiles and the hemodynamic responses in the major arteries of the human leg during prolonged whole leg heating and the subsequent recovery, and during isolated upper leg and lower leg heating. Second, to establish the relationships among conduit artery hyperemia, local tissue oxygenation, and local hyperthermia. And third, to determine the impact of local hyperthermia on local arterial stiffness and distensibility. It was hypothesized that: (1) local hyperthermia would result in profound and sustained increases in blood flow profiles in the arteries supplying the heated leg/leg segment; (2) no changes in the hemodynamic and temperature profiles would be observed in the control leg/adjacent leg segment; (3) local hyperemia and tissue oxygenation are positively related to regional temperature; and (4) local arterial distensibility would largely remain unchanged during whole leg hyperthermia.

## 2 MATERIALS AND METHODS

### 2.1 Participants

This study consisted of three protocols: (1) whole leg heating; (2) upper leg heating; and (3) lower leg and foot heating. In total, eight healthy men and one healthy woman (mean ± SD: age 28 ± 11 years; height 177 ± 8 cm; mass 79.7 ± 9.1 kg) participated in protocol 1, five healthy men and three healthy women (age 32 ± 14 years; height 174 ± 10 cm; mass 72.7 ± 13.9 kg) in protocol 2, and five healthy men and three healthy women (age 29 ± 11 years; height 176 ± 9 cm; mass 72.3 ± 11.2 kg) in protocol 3. Three participants completed all three protocols whereas four completed two. Prior to the start of the study, informed written consent was obtained from all participants following a detailed written and verbal explanation of the experimental protocol. Participants were considered healthy following the completion of a health questionnaire. All procedures were approved by the Brunel University London Research Ethics Committee and are in agreement with the ethical principles stated in the Declaration of Helsinki (2013). Participants refrained from heavy exercise for 48 h, alcohol consumption for 24 h, and caffeine consumption for 12 h before the commencement of the protocols.

### 2.2 Experimental protocols

For all three protocols, participants were asked to consume their usual breakfast and report to the laboratory between 08h00 and 09h00, whereby they fasted until the completion of the protocol. They were weighed in a semi‐nude state and had their height measured (SECA 798 Scale) and then asked to rest in a supine position on a custom‐built bed within a climate chamber set at 21ºC, where they remained for the entire duration of the study.

**Protocol 1: Effects of prolonged whole leg heating on thermal, hemodynamic, and tissue oxygenation responses**

**FIGURE 1:**
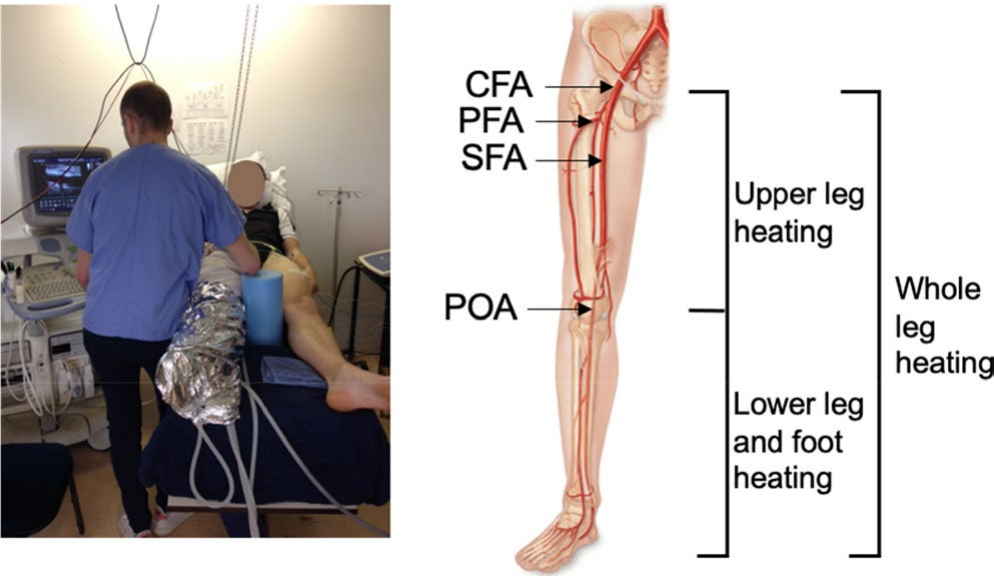
Example of experimental set up. Illustration of the experimental set up—participant was undergoing whole leg heating in the present example. The diagram shows the segments where blood flow was measured for the CFA, SFA, PFA, and POA, respectively, and which segments were heated for the various protocols

Protocol 1 consisted of 3 h of whole leg heating, followed by 3 h of passive recovery. Following instrumentation—ECG electrodes, intravenous cannulation at the antecubital vein, and temperature thermistors (described below)—participants were fitted with a custom‐made water‐perfusion trouser on their right leg, which was then wrapped in a survival blanket to limit heat loss. The trouser was connected to a thermostatically controlled water circulator (Julabo F‐34), which continuously circulated 50ºC water for the first 1.5 h of heating. The water was later reduced to 48ºC to prevent a large increase in core temperature. Blood flow was measured every 30 min in the common (CFA), superficial (SFA) and profunda (PFA) femoral arteries, and popliteal artery (POA) of the experimental and control legs (Figure 1). A 5 ml of venous blood sample and subjective perceptual measures were also collected at the same time points. Hemoglobin and hematocrit concentrations were measured using a commercially available Hb analyzer (Hb 201^+^ system, HemoCue AB), and a microscope and a ruled apparatus were used after centrifuging blood samples for 5 min (Billett, 1990), respectively. Hemoglobin and hematocrit values were then used to calculate blood volume, red blood cell volume, and plasma volume changes and estimate their absolute values using previously described methods (Dill & Costill, 1974; Sawka et al., 1992). Subjective perceptual measures were obtained with a 5‐point thermal comfort scale (*T*_comf_) (1 representing comfortable and 5 very uncomfortable, respectively) (Willmott et al., 2017) and an 8‐point thermal sensation (*T*_sens_) scale (0 representing unbearably cold and 8 unbearably hot, respectively) (Toner et al., 1986).

**Protocol 2: Effects of upper leg heating on thermal, hemodynamic, and tissue oxygenation responses**. Following the instrumentation of ECG electrodes and temperature thermistors, much like protocol 1, participants were fitted with a custom‐made water‐perfusion trouser on their right upper leg, which was then wrapped in a survival blanket to prevent heat loss (Figure 1). The trouser was connected to a thermostatically controlled water circulator, which continuously circulated 50ºC water for the first 1 h of heating, before circulating 20ºC water for the 30 min of cooling, and lastly circulated 50ºC water for the last 1 h of heating. Blood flow was measured every 10 min in the CFA and POA of the experimental leg, and in the control leg at baseline and 150 min of the protocol.

**Protocol 3: Effects of lower leg and foot heating on thermal, hemodynamic, and tissue oxygenation responses**. Following the instrumentation of ECG electrodes and temperature thermistors, much like the previous protocols, participants were fitted with a custom‐made water‐perfusion trouser on their right lower leg and foot, which were then wrapped in a survival blanket to limit heat loss. The trouser was connected to a thermostatically controlled water circulator, which continuously circulated 50ºC water for 1 h of heating. Blood flow was measured every 20 min in the CFA, SFA, PFA, and POA of the experimental leg (Figure 1), and in the control leg at baseline and 60 min of the protocol.

### 2.3 Temperature measurements

Core temperature (*T*_c_) was measured using a rectal probe (Ret‐1 Special, Physitemp) which was self‐inserted 15 cm past the sphincter muscle. Skin temperature (*T*_sk_) in the thigh and calf for both the experimental and control legs was measured using commercially available thermistors (IT‐18, Physitemp) which were securely held in place using medical tape. Muscle temperature (*T*_m_) in the vastus lateralis muscle and in the gastrocnemius muscle of the experimental and control legs in protocol 1, and only of the experimental leg in protocols 2 and 3, was measured using thermistors (T‐204f, Physitemp) inserted via an 18G catheter ~3 cm into the mid‐portion of the muscle. *T*_c_, *T*_m_, and *T*_sk_ were recorded online using a commercially available thermocouple meter (TC‐2000, Sable Systems International) connected to a data acquisition system (PowerLab 26T, ADInstruments). Additionally, mean leg temperature (${\bar{T}}_{\text{Leg}}$) was calculated as a weighted average: ${\bar{T}}_{\text{Leg}}=\left(T_{\text{m thigh}}\times 0.66\right)+\left(\frac{T_{\text{sk thigh}}+T_{\text{sk hamstring}}}{2}\times 0.06\right)+\left(T_{\text{m calf}}\times 0.25\right)+\left(T_{\text{sk calf}}\times 0.03\right)$; mean upper leg temperature (${\bar{T}}_{\text{Upper leg}}$) was calculated as: ${\bar{T}}_{\text{Upper leg}}=(T_{\text{m thigh}}\times 0.92)+\left(\frac{T_{\text{sk thigh}}+T_{\text{sk hamstring}}}{2}\times 0.08\right)$; and mean lower leg temperature (${\bar{T}}_{\text{Lower leg}}$) was calculated as: ${\bar{T}}_{\text{Lower leg}}=(T_{\text{m calf}}\times 0.88)+\left(T_{\text{sk calf}}\times 0.12\right)$. Mean leg and segmental leg temperature formulas were created using previously reported volume ratios of the different tissue compartments in the leg (Wang et al., 1999).

### 2.4 Hemodynamic measurements

Heart rate was continuously measured using a three‐lead echocardiogram. Moreover, arterial blood pressure, stroke volume, and cardiac output were measured non‐invasively—at the same time points as the arterial blood flow measurements—using infrared photoplethysmography (Finometer, FMS, Netherlands), through a cuff on the middle finger of the right hand. Cardiac output was calculated as heart rate × stroke volume, where stroke volume was directly estimated using the Modelflow method, which incorporated corrections for age, height, and weight (BeatScope, FMS) (Wesseling et al., 1993). Blood flow was measured at set time points—recording two 12 s Doppler scans—throughout the protocols in the various arteries using a duplex Doppler ultrasound system (Vivid 7 Dimension, 198 GE Medical) with a 10 MHz linear array transducer probe (GE Medical Systems) at an insonation angle of ≤60º, with sample volume positioned in the center of the artery. The water‐perfusion heated trouser had custom‐made openings which allowed the probe to be placed on the skin with minimal heat loss. Before commencing baseline blood flow measures, arterial sites for the CFA, SFA, PFA, and POA in both legs were located and marked to ensure that blood flow measures were consistently measured at the same site. SFA and PFA blood flow measurements were acquired at a distance of ≥2 cm from the femoral bifurcation to prevent turbulent flow disruption to the measurements, and thus improve the validity of measures. Blood flow (ml min^−1^) was calculated using the following equation: $BF=V_{\text{mean}}\times \pi \times {\left(\frac{D_{\text{mean}}}{2}\right)}^{2}\times 60$, where *V*_mean_ is the average blood velocity (cm s^−1^) and *D*_mean_ (cm) is the average diameter calculated using: $D_{\text{mean}}=\frac{1}{3}\left(D_{\text{systole}}\right)+\frac{2}{3}\left(D_{\text{diastole}}\right)$ (Rådegran, 1997). Moreover, the arterial diameter was determined using CAROLAB (Zahnd et al., 2013) which was a prerequisite for the assessment of arterial distensibility and wave intensity parameters—outlined in the subsequent section. CAROLAB (Zahnd et al., 2013) uses block matching to provide an accurate measurement of the vessel diameter at each frame. A comparison of arterial diameter between the two methods was performed to ensure validity and reliability. On average, the continuous arterial wall tracking software revealed diameter values 1.9 ± 2.0% (*p* < 0.0001) higher than those obtained via the previously described weighted average of peak systolic and diastolic diameter. Additionally, blood flow was expressed in terms relative to tissue mass—that is, ml min^−1^ 100 g^−1^—and was calculated using the participants’ body mass and previously reported segment mass to body mass ratios (Clauser et al., 1969).

Shear rate (SR) was calculated using: $\text{SR}=\frac{4\times V_{\text{mean}}}{D_{\text{mean}}}$, where *V*_mean_ is the mean blood velocity. Additionally, vascular conductance (VC) was calculated using the following formula: VC = BF÷MAP, where it is represented as ml min^−1^ mmHg^−1^, BF is blood flow (ml min^−1^), and MAP is the mean arterial pressure (mmHg). Blood flow was analyzed offline using commercially available software (EchoPAC, GE Medical). Blood velocity was averaged over two 12 s Doppler scans and average diameter was determined from four 2D B‐mode images. Furthermore, blood pressure and temperature data were collected at 1000 Hz using a commercially available data acquisition system (PowerLab 26T, ADInstruments) and exported in 1 min bins using a commercially available data acquisition software (LabChart 7, ADInstruments). Following exportation, data were imported and analyzed in Microsoft Excel software (Microsoft Corporation). Data are reported as 2 min averages throughout the three protocols. Additionally, quadriceps skin blood flow was measured in all three leg heating protocols via laser‐Doppler flowmetry (PeriFlux Flowmetry System), reported in perfusion units (PU). The probe was attached to the skin of the thigh, specifically on the vastus lateralis.

### 2.5 Wave intensity and local arterial distensibility

Following the obtainment of the ultrasound B‐mode scans, as described above, images were exported as DICOM files for offline analysis. Wave speed calculation and wave intensity analysis were only performed on the CFA of both the experimental and control legs as the DICOM image quality for the other arteries, particularly that of the PFA and POA, was not sufficient for diameter block matching. Diameter waveform extraction was performed using CAROLAB (Zahnd et al., 2013), which uses block matching to provide an accurate measurement of the vessel diameter at each frame. Extracted diameter waveforms were saved as Excel files (Microsoft Corporation) for later analysis. Doppler ultrasound DICOM files were analyzed in MATLAB (version R2019b, The MathWorks, Inc.) to extract the flow velocity waveforms, using custom‐designed algorithms as previously reported (Negoita et al., 2018). Diameter waveform data were obtained for the CFA every 30 min.

These diameter and flow velocity waveforms were then used to calculate wave speed (*c*) using the ln(*D*)*U*‐loop method (Feng & Khir, 2010); the following equation was used: $c=\pm \frac{1}{2}\frac{dU_{\pm }}{d{\left(\text{ln}D\right)}_{\pm }}$ where dU and $d\left(\text{ln}D\right)$ are the incremental differences between adjacent data of velocity $\left(U\right)$ and diameter $\left(D\right)$. Moreover, forward compression waves (FCW) and forward expansion waves (FEW), which reflect left ventricular performance in early and late systole, respectively, were calculated using previously documented techniques (Pomella et al., 2018). Data outputs were averaged over two scans for the same time point, with three waveforms analyzed per scan. Subsequently, with the determination of *c*, distensibility $\left(D_{s}\right)$ was calculated using the following Bramwell and Hill (1922) equation: $D_{s}=p^{‐1}\times c^{‐2}$, where p represents blood density which was assumed equal to 1050 kg m^−3^ (Pomella et al., 2018).

### 2.6 Tissue oxygenation measures

Direct and continuous measures of regional tissue hemoglobin (venous) oxygen saturation (% rSO_2_) were obtained in the experimental and control legs using two near‐infrared spectroscopy units with four optode pads each (NIRS; INVOS 5100C Cerebral Oximeter; Somanetics Corp). The optode pads were placed on the skin surrounding the quadriceps, hamstrings, calf, and foot of both the control and experimental legs and taped to reduce interference from external light sources.

### 2.7 Statistical analysis

Statistical analysis was conducted using R (version 3.5.1, Team [2013]). For protocol 1, linear mixed‐effects models (two‐way) were performed to investigate differences in hemodynamics, flow profiles, and temperature between and within the experimental and control legs over time. In addition, linear mixed‐effects models (one‐way) were conducted to investigate differences over time in systemic variables—that is, heart rate, cardiac output, stroke volume, blood pressure, perceptual measures, and non‐leg temperatures. For protocols 2 and 3, linear mixed‐effects models (one‐way) were conducted to investigate differences over time in all variables—that is, hemodynamics, flow profiles, and temperature. Following the linear mixed‐effects models, once a significant effect was found, a Tukey's *post hoc* test was conducted to locate the specific time points at which those changes occurred. Additionally, linear, exponential, and polynomial regression curve fit tests were performed using GraphPad Prism (version 8, GraphPad Software) to assess the relationship between various key data. Subsequently, Akaike's Information Criterion was used to evaluate which model provides the most appropriate fit. Where an exponential curve fit was appropriate, the equation $y=y_{0}\cdot e^{‐K\cdot x}$ was used, where *y*_0_ is the *y* value when *x* (time) is zero and *k* is the rate constant. Significance of this fit is reported through 95% confidence intervals on the estimated value of *k* with the null value being *k* = 0. Moreover, a MANOVA test was conducted using SPSS (Version 26; IBM) to compare the difference in blood flow between the three heating protocols. After, if a significant effect was found, a Bonferroni's *post hoc* test was conducted to locate the specific time points at which those changes occurred.

## 3 RESULTS

### 3.1 Protocol 1: Effects of prolonged whole leg heating on thermal, hemodynamic, and tissue oxygenation responses

**Regional and core temperatures, and thermal perception**

**FIGURE 2:**
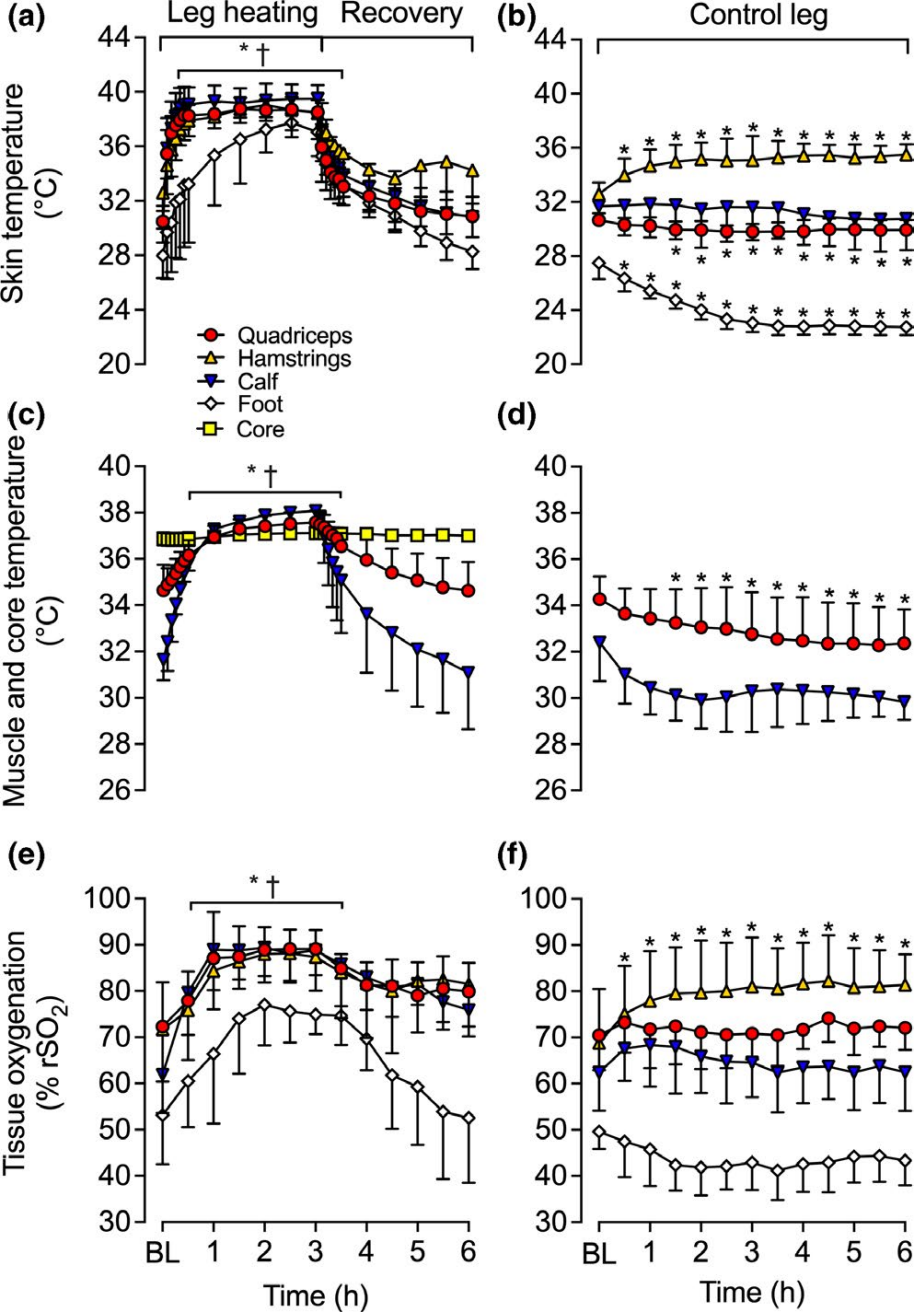
Core and leg temperatures (a, b, c, d) and regional tissue oxygenation (e, f) during whole leg hyperthermia and recovery in the experimental (heated; a, c, e) and control (contralateral; b, d, f) legs. Data represented as mean ± SD (*n* = 9). BL signifies baseline measurements. ∗ Different from baseline, *p* < 0.05. † Different from respective control (contralateral) leg, *p* < 0.05. All variables below the half‐tick down line reported significant differences

Full leg and core temperature responses are illustrated in Figure 2. As per the design, quadriceps, hamstring, calf and foot *T*_sk_, and quadriceps and calf *T*_m_, and thus, ${\bar{T}}_{\text{Leg}}$ of the experimental leg increased progressively during the 3 h whole leg heating protocol, whereas it remained unchanged or declined in the control leg (Figure 2). Specifically, experimental ${\bar{T}}_{\text{Leg}}$ increased by 3.6 ± 0.3ºC (*p* < 0.0001) following 3 h of heating, while the control ${\bar{T}}_{\text{Leg}}$ steadily declined throughout the protocol (∆ = −2.2 ± 1.5ºC, *p* < 0.0001). Experimental ${\bar{T}}_{\text{Leg}}$ remained elevated during the first 1 h of recovery (*p* = 0.008), before declining toward baseline. However, *T*_c_ was only significantly elevated at 3 h (0.2 ± 0.2ºC; *p* < 0.0001), reaching a peak temperature of 37.1 ± 0.2ºC (Figure 2). Perceptual responses—thermal comfort (*T*_comf_) and thermal sensation (*T*_sens_)—remained low and stable throughout the 6 h protocol. *T*_comf_ averaged 1.2 ± 0.8 units (*p* = 0.214), while *T*_sens_ averaged 4.4 ± 0.7 units (*p* = 0.041) increasing by 1 unit between 1.5 and 2.5 h of whole leg heating (*p* = 0.029).

**Leg blood flow, tissue oxygenation, and systemic hemodynamics**

**FIGURE 3:**
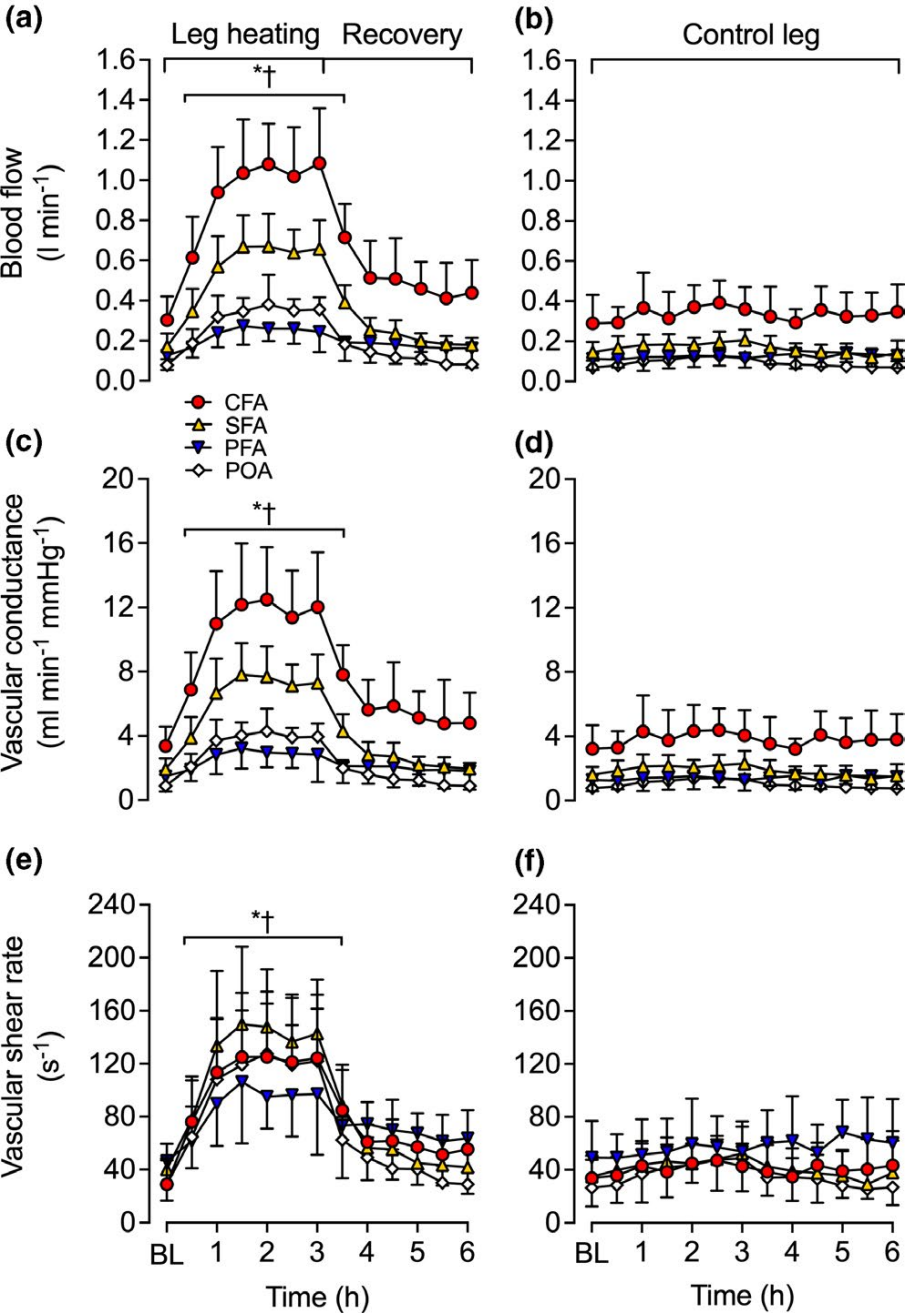
Blood flow (a, b), vascular conductance (c, d), and shear rate (e, f) during whole leg hyperthermia and recovery in CFA, SFA, PFA, and POA of the experimental (heated; a, c, e) and control (contralateral; b, d, f) legs. Data represented as mean ± SD (*n* = 9). BL signifies baseline measurements. ∗ Different from baseline, *p* < 0.05. † Different from respective control (contralateral) leg, *p* < 0.05. All variables below the half‐tick down line reported significant differences

Complete blood flow responses for the CFA, SFA, PFA, and POA are illustrated in Figure 3a. In the experimental leg, CFA, SFA, and POA blood flow increased ≥3‐fold and PFA blood flow increased ~2‐fold within the first hour of whole leg heating (*p* < 0.0001) (Figure 3a). Thereafter, arterial blood flow remained elevated and plateaued around 1.5–2 h until the cessation of heating (Figure 3a). During the subsequent 3 h recovery period, blood flow in the CFA, SFA, and POA remained elevated for the first 30 min (CFA and SFA: *p* < 0.0001; POA: *p* = 0.010) and then steadily decreased toward baseline values (Figure 3a). No changes in blood flow were observed in control leg for all four arteries throughout the entirety of the 6 h protocol (*p* > 0.05) (Figure 3b).

In line with the previously mentioned arterial responses, average upper and lower leg blood flow were ~3‐fold higher in the experimental leg than the control leg following the whole leg heating protocol (*p* < 0.0001). Additionally, upper leg blood flow was higher than lower leg blood flow at all times (*p* < 0.0001). On average, experimental upper leg blood flow was 305 ± 249 ml min^−1^ higher than experimental lower leg blood flow during heating. However, when accounted for estimated tissue mass, upper and lower leg blood flow were similar following 3 h of whole leg heating (9.3 ± 2.8 vs. 10.5 ± 2.0 ml min^−1^ 100 g^−1^, *p* = 0.369). Similar responses were observed in shear rate and vascular conductance in all four arteries of the experimental leg (Figure 3c and 3e). Experimental leg CFA, SFA, PFA, and POA shear rate and vascular conductance increased during the whole leg heating protocol, with ~4‐fold increase being observed in the CFA, SFA, and POA at 3 h (*p* < 0.0001), while a ~ 2‐fold increase was observed in the PFA (*p* < 0.0001). In contrast, no changes were observed in the control leg (Figure 3d and 3f). Moreover, whole leg heating increased upper leg tissue oxygenation by 16 ± 9% rSO_2_ units (*p *< 0.0001), and lower leg tissue oxygenation by 24 ± 9% rSO_2_ units (*p* < 0.0001) of the experimental leg (Figure 2e). Furthermore, tissue oxygenation remained unchanged in the control leg (*p* > 0.05, respectively) with the exception of hamstring tissue oxygenation which increased by 12 ± 7% rSO_2_ units at 6 h (*p* < 0.0001; Figure 2f). However, when all the control leg sites were evaluated together, a close linear relationship was observed between the changes in local temperature and tissue oxygenation (*R* ^2^ = 0.88, *p* < 0.0001).

**FIGURE 4:**
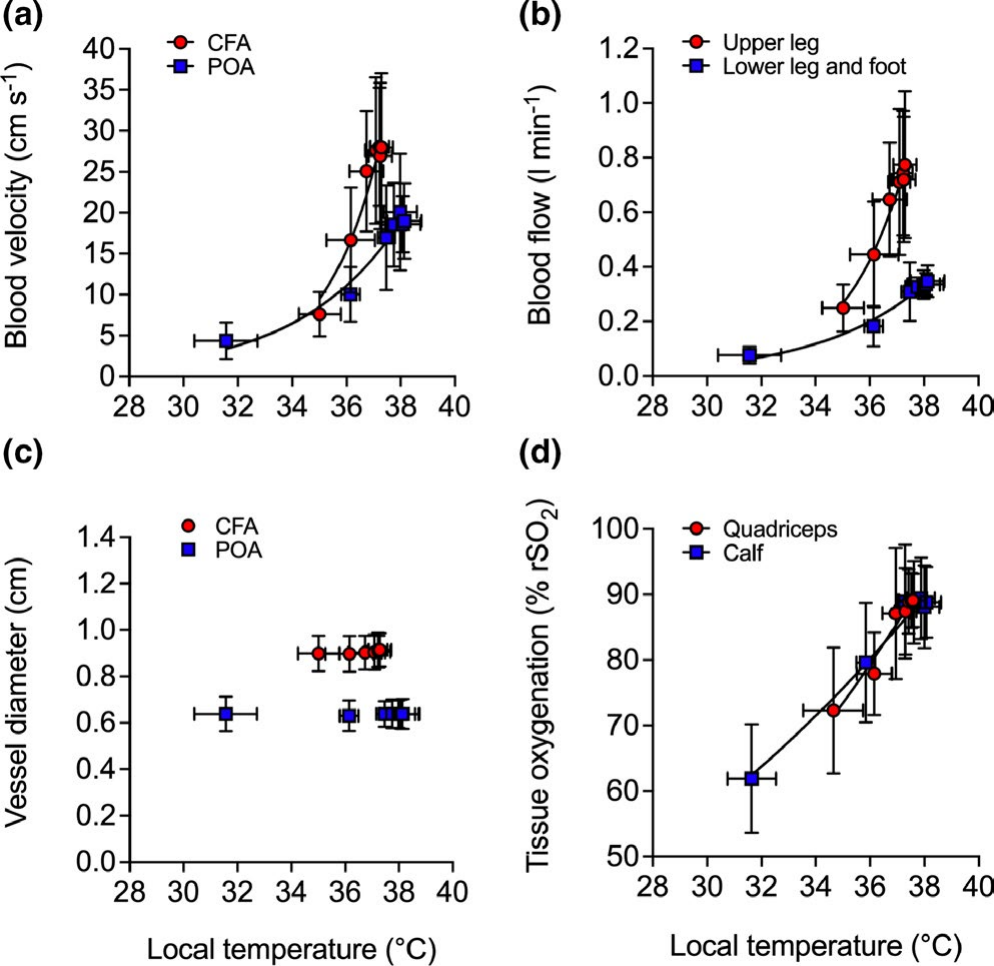
Relationship between the local temperature (${\bar{T}}_{\mathrm{Leg}}$) and local blood velocity (a), blood flow (b), vessel diameter (c), and tissue oxygenation (d) values during whole leg hyperthermia and recovery. Data represented as mean ± SD (*n* = 9). Vertical error bars signify the dependent variable SD, while horizontal error bars signify ${\bar{T}}_{\mathrm{Leg}}$ SD, respectively

Increases in arterial blood flow were exponentially related to increases in local temperature (upper leg: *R* ^2^ = 0.98, *k* = 0.46 [0.37,0.57]; lower leg: *R* ^2^ = 0.98; *k* = 0.26 [0.20,0.35]) (Figure 4b) and were attributed to an increased blood velocity (all *p* ≤ 0.012) (Figure 4a), as arterial diameter remained constant throughout the heating protocol (*p* > 0.1) (Figure 4c). In agreement with the global blood flow dynamic responses, increases in regional tissue oxygenation were exponentially related to increases in local temperature (upper leg: *R* ^2^ = 0.96, *k* = 0.08 [0.06,0.10]; lower leg: *R* ^2^ = 0.98, *k* = 0.06 [0.05,0.07]) (Figure 4d). Experimental leg quadriceps skin blood flow increased during whole leg heating from 3 ± 2 to 78 ± 37 PU (*p* = 0.006) before slowly returning to baseline (3 ± 1 PU) following 3 h of recovery (*p* = 1.00). At the systemic level, no significant changes were observed for systemic blood volume, red cell volume, plasma volume, and cardiac stroke volume (all *p* > 0.5; Table 1). However, increases of 8 ± 7 bpm in heart rate (*p* = 0.002) and 1.2 ± 0.7 L min^−1^ in cardiac output (*p* = 0.038) were observed at 3 h of whole leg heating (Table 1).

**TABLE 1:** Influence of prolonged whole leg heating and subsequent recovery on central hemodynamics, common femoral artery blood flow, wave speed, arterial distensibility, and wave intensity parameters and popliteal artery blood flow

Variables	Intervention	Time (h)						
		Baseline	1	2	3	4	5	6
MAP (mmHg)		89 ± 7	88 ± 10	88 ± 9	91 ± 9	91 ± 8	90 ± 4	92 ± 5
$\dot{Q}$ (L min^−1^)		6.3 ± 0.6	6.8 ± 1.0	7.0 ± 0.8	7.5 ± 0.7^*^	6.7 ± 1.3	6.7 ± 1.7	6.7 ± 1.0
SV (ml)		111 ± 24	107 ± 19	111 ± 24	118 ± 30	116 ± 35	115 ± 41	114 ± 39
HR (beats min^−1^)		58 ± 10	64 ± 8^*^	65 ± 10^*^	66 ± 13^*^	60 ± 10	61 ± 11	63 ± 14
CFA blood flow (ml min^−1^)	Heated leg	320 ± 98	940 ± 226^*†^	1080 ± 202^*†^	1122 ± 250^*†^	513 ± 184	459 ± 134	438 ± 163
	Control leg	289 ± 142	365 ± 177	371 ± 108	360 ± 110	293 ± 67	323 ± 121	347 ± 136
POA blood flow (ml min^−1^)	Heated leg	78 ± 30	319 ± 106^*†^	382 ± 147^*†^	356 ± 60^*†^	145 ± 23	112 ± 33	82 ± 22
	Control leg	70 ± 34	101 ± 58	110 ± 40	120 ± 23	84 ± 19	74 ± 18	70 ± 30
Wave speed (m s^−1^)	Heated leg	20.4 ± 7.8	21.6 ± 10.6	20.6 ± 8.1	21.4 ± 10	22.1 ± 9.1	18.2 ± 6.2	19.3 ± 5.8
	Control leg	16.5 ± 9.3	16.1 ± 7.3	16.4 ± 6.3	16.9 ± 9.1	17.2 ± 3.4	15.4 ± 2.4	17.2 ± 4.4
Distensibility (×10^−3^ mmHg^−1^)	Heated leg	0.3 ± 0.2	0.3 ± 0.3	0.3 ± 0.3	0.3 ± 0.2	0.2 ± 0.2	0.3 ± 0.1	0.2 ± 0.1
	Control leg	0.3 ± 0.2	0.3 ± 0.3	0.3 ± 0.2	0.4 ± 0.2	0.2 ± 0.1	0.3 ± 0.1	0.3 ± 0.1
FCW (cm^2^ s^−1^)	Heated leg	1.6 ± 1.1	2.1 ± 0.8	2.5 ± 0.8	2.0 ± 0.7	1.9 ± 0.7	2.2 ± 0.9	2.6 ± 1.5
	Control leg	2.0 ± 0.8	2.2 ± 0.6	2.5 ± 1.1	1.8 ± 0.5	1.6 ± 0.6	1.7 ± 0.4	1.9 ± 1.0
FEW (cm^2^ s^−1^)	Heated leg	0.5 ± 0.3	0.7 ± 0.3	0.7 ± 0.3	0.7 ± 0.3	0.8 ± 0.2	0.8 ± 0.4	0.8 ± 0.4
	Control leg	0.5 ± 0.3	0.6 ± 0.3	0.6 ± 0.3	0.7 ± 0.4	0.4 ± 0.1	0.6 ± 0.2	0.4 ± 0.2

Values are mean ± SD for nine participants, except for the wave intensity‐derived parameters (n = 6). MAP: mean arterial pressure; $\dot{Q}$: cardiac output; SV: stroke volume; HR: heart rate; CFA: common femoral artery; POA: popliteal artery; FCW: forward compression waves; FEW: forward expansion waves. First 3 h represent whole leg heating responses, while hours 4 to 6 represent responses during the subsequent 3 h passive recovery.

∗ Different from baseline, *p* < 0.05.

† Different from respective control (contralateral) leg, *p* < 0.05.

### 3.2 Protocol 2: effects of upper leg heating on thermal, hemodynamic, and tissue oxygenation responses

**Regional temperature responses**

**FIGURE 5:**
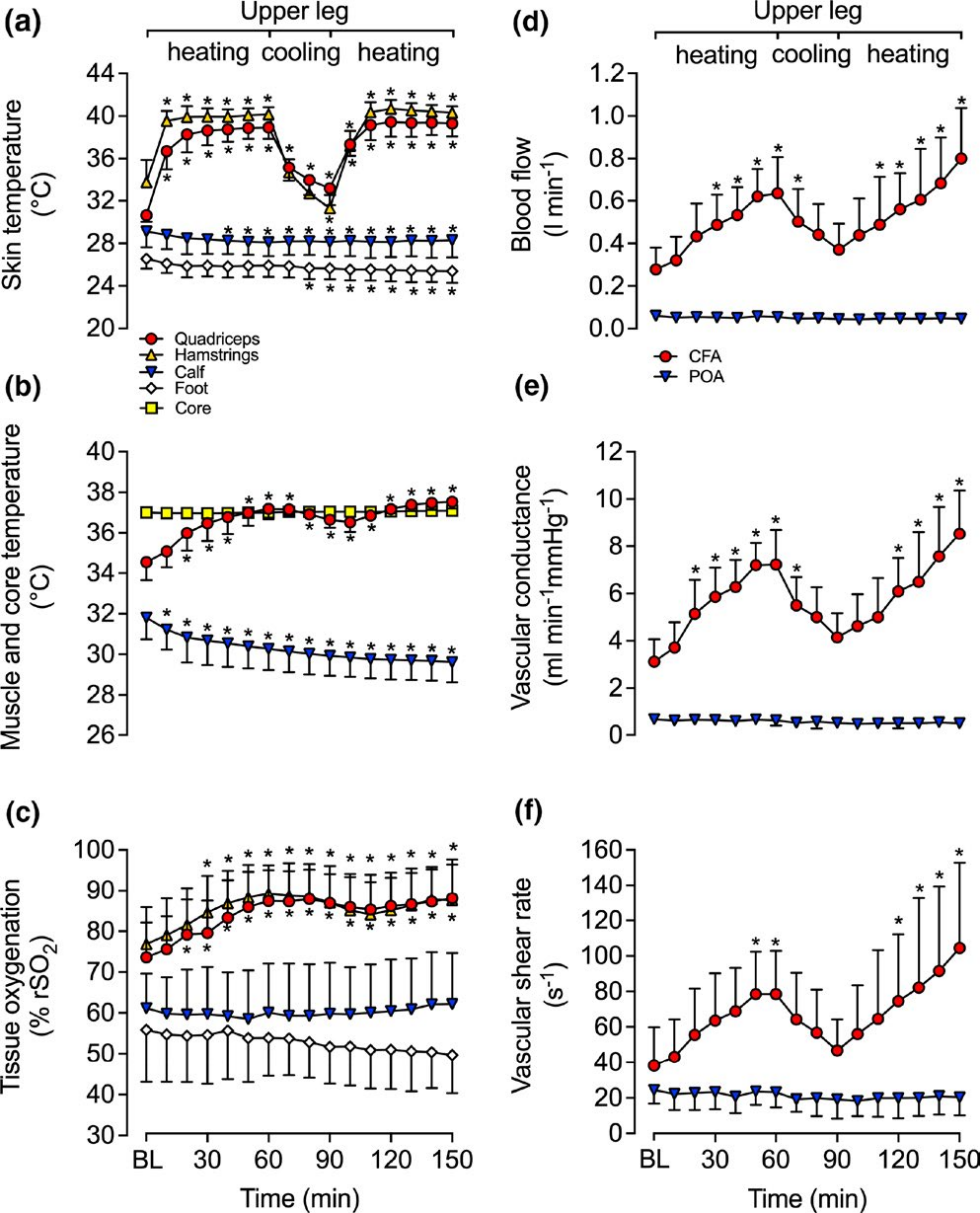
Core and leg temperatures (a, b), regional tissue oxygenation (c), blood flow (d), vascular conductance (e), and shear rate (f) in CFA and POA during upper leg heating, cooling and heating. Data represented as mean ± SD (*n* = 8). BL signifies baseline measurements. ∗ Different from baseline, *p* < 0.05

Full leg and core temperature responses are illustrated in Figure 5. As per the design, substantial increases in quadriceps and hamstrings *T*_sk_, quadriceps *T*_m_ and ${\bar{T}}_{\text{Upper leg}}$ were observed following the two bouts of upper leg heating (all *p* < 0.0001). During the 30 min bout of upper leg cooling, however, upper leg *T*_sk_ dropped with hamstring *T*_sk_ declining below baseline (∆ = −2.4 ± 2.5ºC, *p* = 0.0004) whereas quadriceps *T*_sk_ remained elevated from baseline (∆ = +2.6 ± 1.7ºC, *p* = 0.002) (Figure 5a). Similar to quadriceps *T*_sk_, *T*_m_ remained elevated above baseline during the cooling bout (∆ = +2.1 ± 1.0ºC, *p* = 0.0001) (Figure 5b). Consequently, ${\bar{T}}_{\text{Upper leg}}$ was elevated throughout the entirety of the protocol, increasing from 34.4 ± 0.8ºC to 37.7 ± 0.4ºC at 150 min (*p* < 0.0001). In contrast, in the lower leg, calf *T*_sk_ tended to decline (*p* = 0.054), while calf *T*_m_ and foot *T*_sk_ decreased −2.2 ± 0.4ºC and −1.2 ± 1.0ºC, respectively (both *p* < 0.0001) at 150 min. *T*_c_ and control leg quadriceps *T*_sk_ remained unaltered throughout the protocol (*p* = 0.105 and *p* = 0.214, respectively) (Figure 5a and 5b).

**Leg blood flow, tissue oxygenation, and systemic hemodynamics**

**FIGURE 6:**
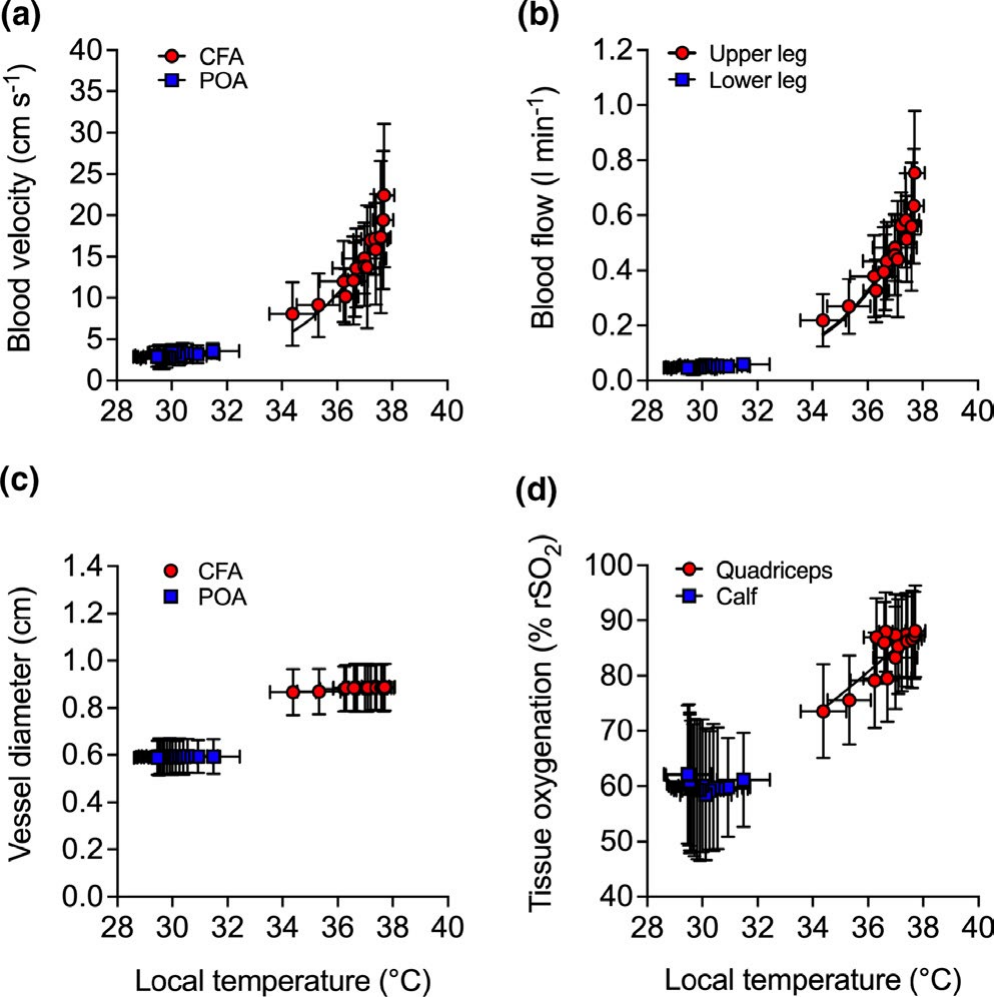
Relationship between the local temperature (${\bar{T}}_{\mathrm{Leg}}$) and local blood velocity (a), blood flow (b), vessel diameter (c), and tissue oxygenation (d) values during upper leg heating and cooling. Data represented as mean ± SD (*n* = 8). Vertical error bars signify the dependent variable SD, while horizontal error bars signify ${\bar{T}}_{\mathrm{Leg}}$ SD, respectively

Complete blood flow responses for the CFA and POA are illustrated in Figure 5d. Following the first bout of 1 h upper leg heating, CFA blood flow increased 2.3‐fold (*p* < 0.0001) and then declined toward baseline during the 30 min bout of upper leg cooling (*p* = 0.639) (Figure 5d). In the second bout of 1 h upper leg heating, CFA blood flow surpassed the magnitude obtained in the first bout of heating—increasing 2.9‐fold and peaking at 801 ± 237 ml min^−1^ (*p* < 0.0001) (Figure 5d). These increases in CFA blood flow were exponentially related to an increasing ${\bar{T}}_{\text{Upper leg}}$ (*R* ^2^ = 0.89, *k* = 0.40 [0.31, 0.51]) (Figure 6b) and were the result of an increased blood velocity (*p* = 0.001) (Figure 6a), as CFA diameter remained unchanged (*p* = 0.065) (Figure 6c). Throughout the protocol, POA blood flow remained stable (*p* = 0.149) (Figure 5d). Accordingly, the calculated upper leg blood flow increased by 536 ± 243 ml min^−1^ following the second 1 h heating bout (*p* < 0.0001). Upper leg blood flow was higher than lower leg blood flow at all time points, ranging from a 3.6‐fold difference at baseline to a 16‐fold difference following the second bout of upper leg heating (*p* < 0.0001). In the control leg, CFA and POA blood flow pre‐ and post‐protocol remained stable (*p* = 0.369 and *p* = 0.150, respectively).

Shear rate and vascular conductance mirrored the blood flow responses in both the CFA and POA: CFA shear rate and vascular conductance increased ~2‐fold in the first bout and almost 3‐fold in the second 1 h bout of upper leg heating (both *p* < 0.0001) (Figure 5). Conversely, POA shear rate and vascular conductance remained stable throughout the entire protocol (shear rate: *p* = 0.153; vascular conductance: *p* = 0.107) (Figure 5e and 5f). Furthermore, quadriceps skin blood flow increased from 7 ± 9 to 58 ± 27 PU during the first 1 h bout of upper leg heating. Quadriceps skin blood flow declined toward baseline at the end of the 30 min upper leg cooling bout (*p* = 0.996 vs. baseline), before increasing to 43 ± 28 PU (*p* = 0.003) once again during the second bout of upper leg heating. Tissue oxygenation responses are illustrated in Figure 5c. At all‐time points, upper leg tissue oxygenation was higher than lower leg tissue oxygenation (*p* < 0.0001). During the first 1 h upper leg heating bout, upper leg tissue oxygenation increased by 13 ± 4% rSO_2_ units (*p* < 0.0001), increasing exponentially in response to ${\bar{T}}_{\text{Upper leg}}$ (*R* ^2^ = 0.71, *k* = 0.05 [0.03,0.07]) (Figure 6d). Subsequently, upper leg tissue oxygenation plateaued and remained elevated during the succeeding upper leg cooling and upper leg heating bouts (*p* < 0.0001) (Figure 5c). Conversely, calf and foot tissue oxygenation remained unchanged (*p* = 0.350 and *p* = 0.074, respectively) (Figure 5c). At the systemic level, heart rate, cardiac output, stroke volume, and mean arterial pressure remained stable with averages of 57 ± 5 bpm, 5.1 ± 0.8 L min^−1^, 91 ± 15 ml, and 89 ± 14 mmHg, respectively (all *p* > 0.05) (Table 2).

**TABLE 2:** Influence of upper leg heating, cooling and heating on central hemodynamics and common femoral and popliteal artery blood flow

Variables	Intervention	Time (min)					
		Baseline	30	60	90	120	150
MAP (mmHg)		88 ± 16	83 ± 14	88 ± 14	88 ± 11	92 ± 12	94 ± 18
$\dot{Q}$ (L min^−1^)		4.9 ± 0.8	5.0 ± 0.7	5.0 ± 0.8	5.2 ± 0.9	5.2 ± 0.7	5.5 ± 1
SV (ml)		89 ± 13	92 ± 15	91 ± 17	93 ± 19	92 ± 16	101 ± 24
HR (beats min^−1^)		57 ± 5	57 ± 6	58 ± 9	57 ± 3	58 ± 5	56 ± 6
CFA blood flow (ml min^−1^)	Heated leg	278 ± 102	487 ± 142^*^	636 ± 170^*^	371 ± 122	561 ± 168^*^	801 ± 237^*^
	Control leg	299 ± 133	—	—	—	—	277 ± 127
POA blood flow (ml min ^−1^)	Heated leg	60 ± 18	53 ± 18	54 ± 18	45 ± 19	47 ± 23	47 ± 18
	Control leg	61 ± 19	—	—	—	—	49 ± 23

Values are mean ± SD for eight participants, except for $\dot{Q}$ and SV values where *n* = 6. MAP: mean arterial pressure; $\dot{Q}$: cardiac output; SV: stroke volume; HR: heart rate; CFA: common femoral artery; POA: popliteal artery. Baseline to 60 min represents upper leg heating responses, 90 min represents upper leg cooling, and 120 min to 150 min represents the second bout of upper leg heating.

∗ Different from baseline, *p* < 0.05.

### 3.3 Protocol 3: effects of lower leg and foot heating on thermal, hemodynamic, and tissue oxygenation responses

**FIGURE 7:**
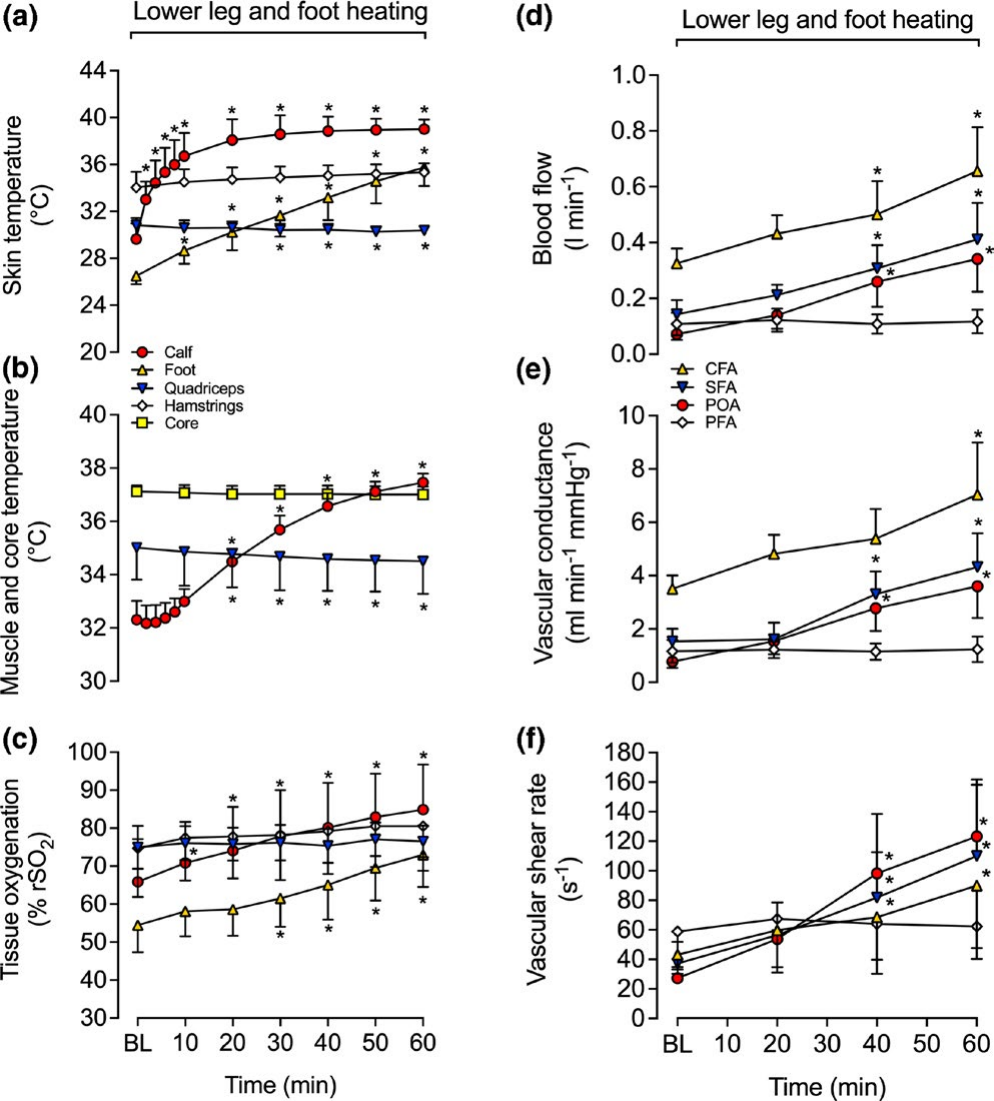
Core and leg temperatures (a, b), regional tissue oxygenation (c), blood flow (d), vascular conductance (e), and shear rate (f) in CFA, SFA, PFA, and POA during lower leg and foot heating. Data represented as mean ± SD (*n* = 8). BL signifies baseline measurements. ^∗^ Different from baseline, *p* < 0.05

**Regional temperature responses**. Full leg and core temperature responses are illustrated in Figure 7. As per the design, substantial increases in calf and foot *T*_sk_, calf *T*_m_ and ${\bar{T}}_{\text{Lower leg}}$ were observed following the 1 h bout of lower leg and foot heating with ${\bar{T}}_{\text{Lower leg}}$ increasing from 32.0 ± 0.7ºC to 37.7 ± 0.3ºC (*p* < 0.0001). In contrast, ${\bar{T}}_{\text{Upper leg}}$ slowly declined throughout the protocol (∆ = −0.4 ± 0.2ºC, *p* = 0.003). *T*_c_ remained unaltered (*p* = 0.081) (Figure 7b).

**FIGURE 8:**
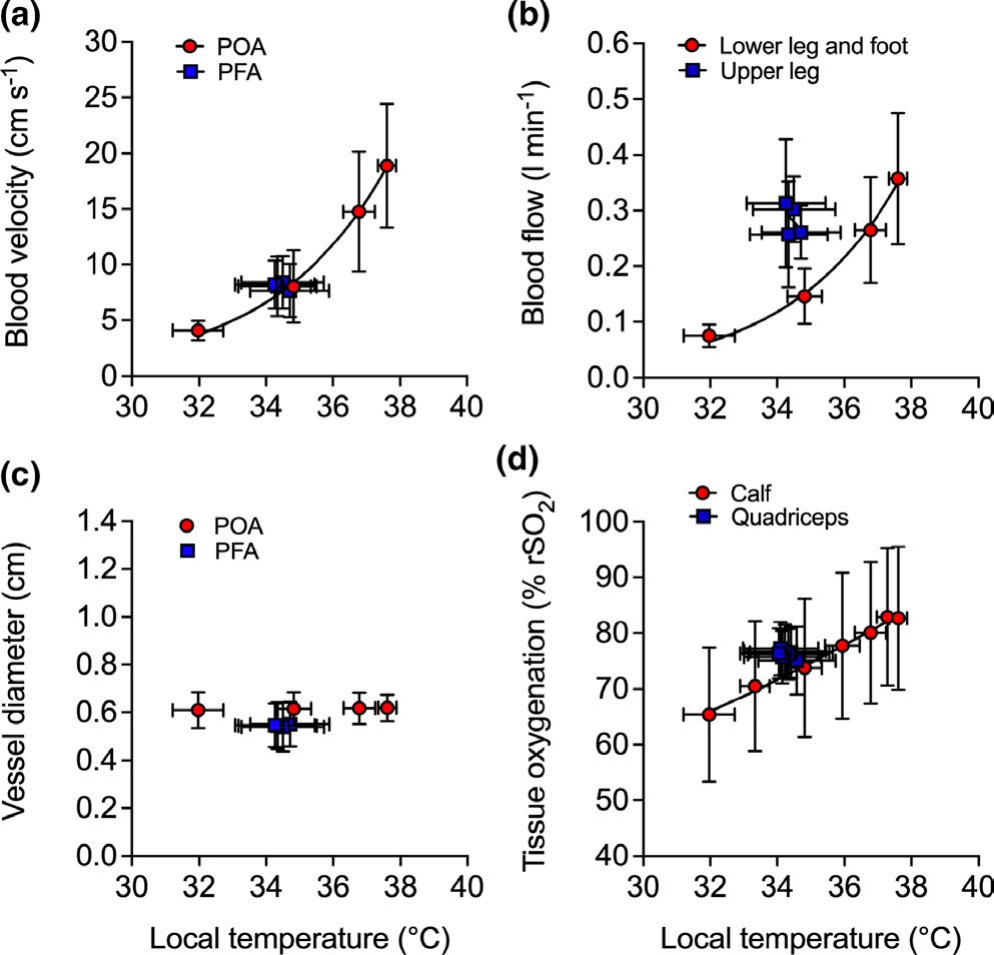
Relationship between the local temperature (${\bar{T}}_{\mathrm{Leg}}$) and local blood velocity (a), blood flow (b), vessel diameter (c), and tissue oxygenation (d) values during lower leg and foot heating. Data represented as mean ± SD (*n* = 8). Vertical error bars signify the dependent variable SD, while horizontal error bars signify ${\bar{T}}_{\mathrm{Leg}}$ SD, respectively

**Leg blood flow, tissue oxygenation, and systemic hemodynamics**. Complete blood flow responses for the CFA, SFA, PFA, and POA are illustrated in Figure 7. In response to lower leg and foot heating, CFA, SFA, and POA blood flow increased, while PFA blood flow remained stable (*p* = 0.474). Following 1 h of lower leg heating, CFA and SFA blood flow increased >2‐fold (*p* < 0.0001) and POA blood flow increased 4.7‐fold (*p* < 0.0001) (Figure 7d). The increase in POA blood flow was exponentially related to increases in ${\bar{T}}_{\text{Lower leg}}$ (*R* ^2^ = 0.99, *k* = 0.30 [0.22,0.41]) (Figure 8b), and was attributed to an increased blood velocity (*p* < 0.001) (Figure 8a) as diameter remained unchanged (*p* > 0.1) (Figure 8c). Upper leg blood flow remained constant throughout the entire protocol at 276 ± 84 ml min^−1^ (*p* = 0.257) as the increases in CFA and SFA blood flow were proportional to the increase in lower leg blood flow. Similarly, CFA, SFA, and POA shear rate and vascular conductance increased during the lower leg and foot heating protocol, with over 2‐fold increases being observed at 1 h in both the CFA and SFA (*p* < 0.0001 for shear rate and vascular conductance in both arteries, respectively), while POA shear rate and vascular conductance increased ~4.5‐fold at 1 h (both *p* < 0.0001). In contrast, no changes in PFA shear rate and vascular conductance were observed (shear rate: *p* = 0.549; vascular conductance: *p* = 0.476). In response to lower leg and foot heating, lower leg and foot tissue oxygenation increased by 18 ± 6% rSO_2_ units, respectively (*p* < 0.0001). These increases in lower leg tissue oxygenation were exponentially related to increases in ${\bar{T}}_{\text{Lower leg}}$ (*R* ^2^ = 0.99, *k* = 0.04 [0.04, 0.05]), respectively (Figure 8d). No changes in quadriceps tissue oxygenation (*p* = 0.210; Figure 7c) or quadriceps skin blood flow (*p* = 0.462) were observed during lower leg and foot heating. However, similar to the control leg during whole leg heating, hamstrings tissue oxygenation increased slightly by 6 ± 3% rSO_2_ units (*p* = 0.015; Figure 7c). At the systemic level, heart rate, cardiac output, stroke volume, and mean arterial pressure remained stable with averages of 59 ± 8 bpm, 5.2 ± 0.9 L min^−1^, 89 ± 18 ml, and 93 ± 43 mmHg, respectively (all *p* > 0.05) (Table 3). In addition, no changes in control leg CFA, SFA, PFA, and POA blood flow pre‐ and post‐protocol were observed (all *p* > 0.05).

**TABLE 3:** Influence of lower leg and foot heating on central hemodynamics and common femoral and popliteal artery blood flow

Variables	Intervention	Time (min)			
		Baseline	20	40	60
MAP (mmHg)		93 ± 10	90 ± 9	93 ± 11	96 ± 18
$\dot{Q}$ (L min^−1^)		5.1 ± 0.9	5.2 ± 0.8	5.0 ± 1.0	5.3 ± 1.1
SV (ml)		89 ± 20	88 ± 15	89 ± 24	89 ± 14
HR (beats min^−1^)		59 ± 8	59 ± 9	58 ± 8	59 ± 8
CFA blood flow (ml min^−1^)	Heated leg	325 ± 53	432 ± 66	502 ± 119^*^	656 ± 157^*^
	Control leg	327 ± 85	—	—	314 ± 39
POA blood flow (ml min^−1^)	Heated leg	72 ± 21	140 ± 49	259 ± 89^*^	341 ± 117^*^
	Control leg	74 ± 22	—	—	64 ± 11

Values are mean ± SD for seven participants, except for blood flow values where *n* = 8. MAP: mean arterial pressure; $\dot{Q}$: cardiac output; SV: stroke volume; HR: heart rate; CFA: common femoral artery; POA: popliteal artery. Baseline to 60 min represents lower leg and foot heating responses.

∗ Different from baseline, *p* < 0.05.

### 3.4 All protocols: comparison of changes in regional blood flow with whole leg, upper leg, and lower leg and foot hyperthermia

**FIGURE 9:**
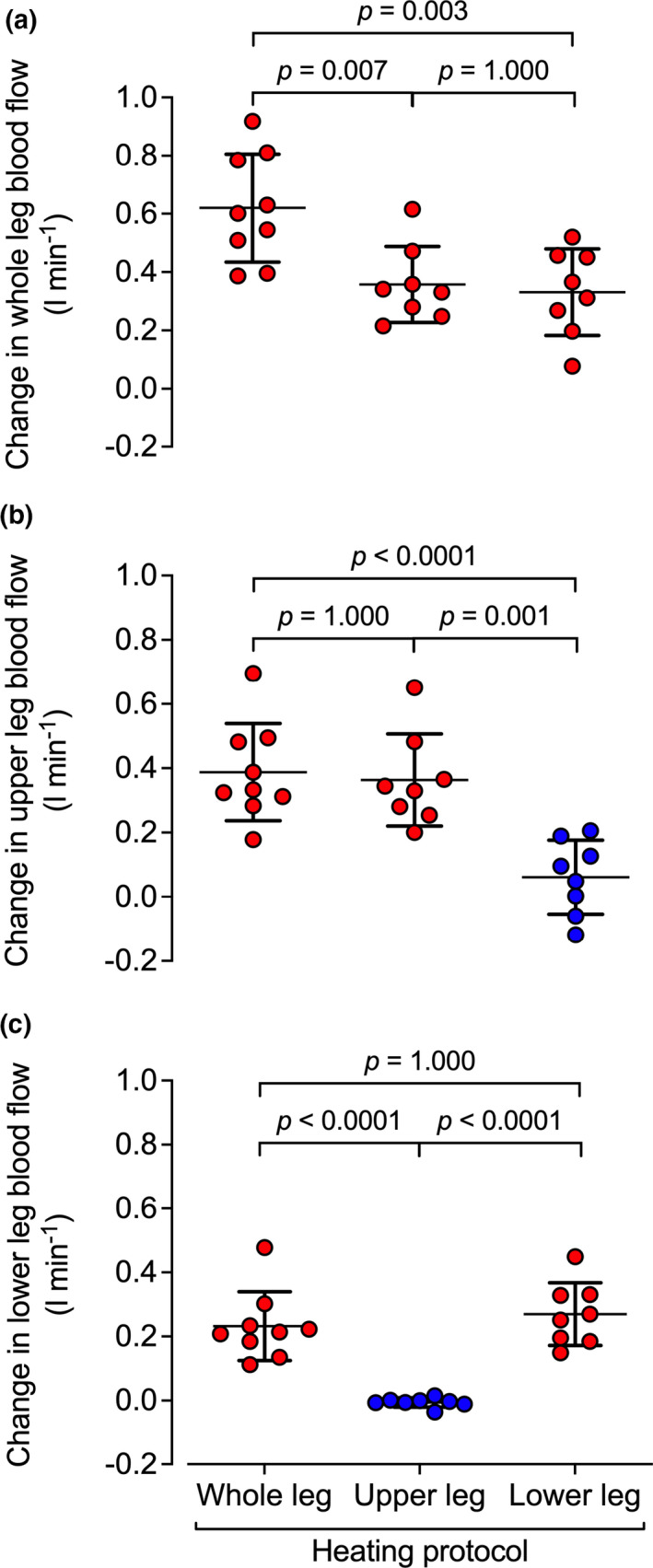
Changes in regional blood flow during 1 h whole leg and segmental leg heating. Circles depict the individual data points while the lines illustrate mean ± SD (*n* = 25). Red circles represent heated segments while blue circles represent control segments, respectively. The figure reports three levels of comparisons: whole leg versus upper leg blood flow, whole leg versus lower leg blood flow, and upper leg versus lower leg blood flow, respectively, with *p* values and half‐tick down lines illustrating the differences. Note that increases in segmental blood flow reflect the changes in local temperature, regardless of the heating protocol

**FIGURE 10:**
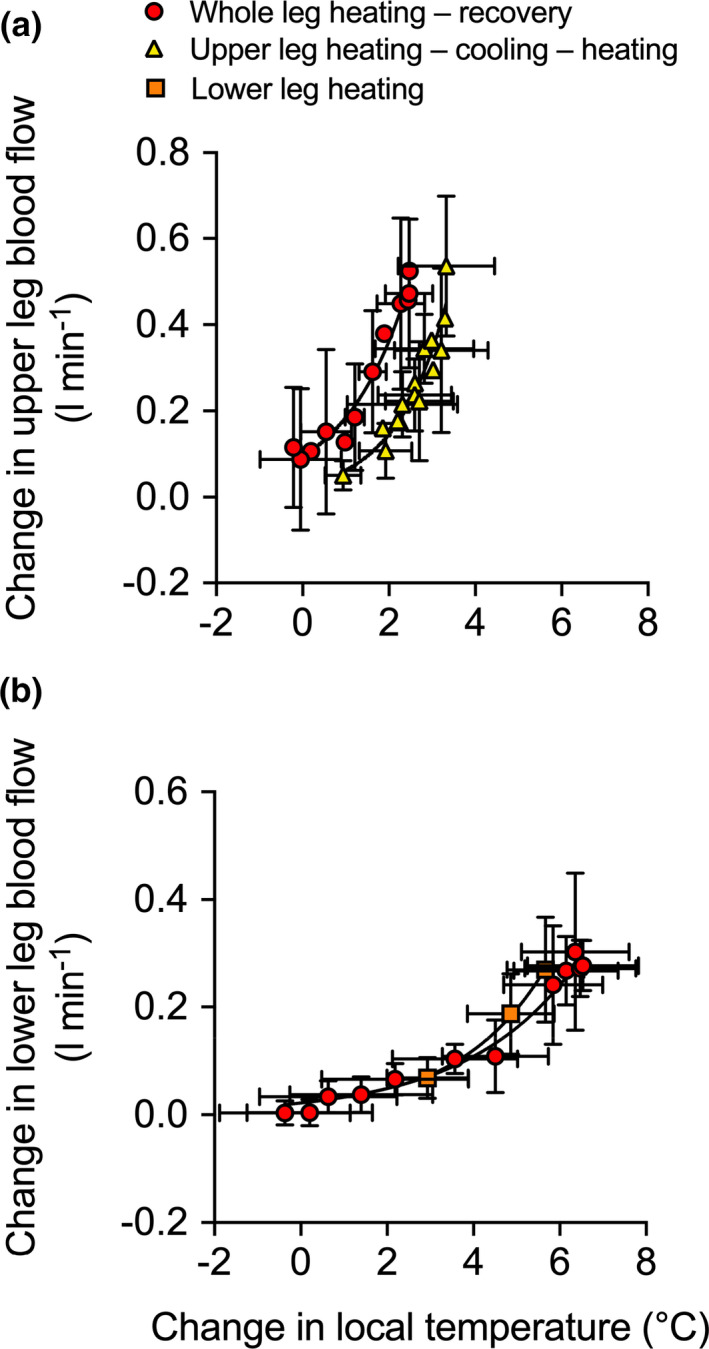
Relationship between the change in local temperature (${\bar{T}}_{\mathrm{Leg}}$) and the change in local blood flow during whole leg, upper leg, and lower leg and foot heating. Data represented as mean ± SD (*n* = 25). Vertical error bars signify ∆ blood flow SD, while horizontal error bars signify ∆ ${\bar{T}}_{\mathrm{Leg}}$ SD, respectively

Individual and mean changes in whole leg, upper leg, and lower leg blood flow during the first hour of the three heating protocols are illustrated in Figure 9. The increase in whole leg blood flow was greater following 1 h of whole leg heating than during 1 h of upper‐ (*p* = 0.007) and lower leg heating *(p* = 0.003); however, upper leg and lower leg blood flow were not different from one another during whole leg heating (*p* = 0.155). During the upper leg heating protocol, the increase in whole leg blood flow was not different from the increase in upper leg blood flow (*p* = 1.00) but the elevation in upper leg blood flow was higher than that of lower leg blood flow (*p* < 0.0001). Last, during lower leg and foot heating, the increase in lower leg blood flow was similar to the increase in whole leg blood flow (*p* = 0.982), such that the change in lower leg blood flow was greater than that of upper leg blood flow (*p* = 0.008). Moreover, these changes in upper leg and lower leg blood flow occurred exponentially to the change in temperature (Figure 10). Strong relationships between the change in upper leg blood flow and the change in upper leg temperature exist during whole leg heating (*R* ^2^ = 0.97, *k* = 0.67 [0.57,0.79]) and upper leg heating (*R* ^2^ = 0.89, *k* = 0.85 [0.65,1.08]). Similarly, strong exponential relationships between the change in lower leg blood flow and the change in lower leg temperature exist during whole leg heating (*R* ^2^ = 0.98, *k* = 0.40 [0.33,0.49]) and lower leg and foot heating (*R* ^2^ = 0.99, *k* = 0.49 [0.21,0.94]).

### 3.5 Wave speed, local arterial distensibility, and wave intensity parameters

Wave speed, distensibility, and wave intensity parameters measured at the CFA during prolonged whole leg heating and its subsequent recovery are reported in Table 1. Wave speed remained stable throughout the protocol in both legs (*p* = 0.908) with no differences being observed between the experimental and control leg (*p* = 0.324). Consequently, as arterial distensibility is calculated from wave speed, no changes were observed throughout the protocol (*p* = 0.841) or between legs (*p* = 0.329). Similarly, wave intensity parameters, forward compression, and forward expansion waves did not change in response to whole leg heating (*p* = 0.218 and 0.860, respectively), nor were there any differences between legs (*p* = 0.371 and 0.097, respectively).

## 4 DISCUSSION

This study explored the relationships between local hyperthermia and the hemodynamic profiles of the leg major arteries and the oxygenation of the tissues they perfuse, comparing the responses of experimental and control legs as well as the upper and lower leg segments during prolonged whole leg and segmental leg heating. In line with the study's hypotheses, local macro‐ and microvascular blood flows were closely related to local temperature across all the experimental conditions producing large variations in local temperature, but essentially no changes in core temperature. Whole leg hyperthermia markedly increased blood flow and vascular conductance in the four major arteries during the 3 h heating protocol, then slowly declined during the subsequent recovery in association with the fall in local temperature. Segmental leg hyperthermia elicited comparable increases in regional blood flow to that of the regional hyperemia observed during whole leg hyperthermia. Additionally, increases in blood flow of the heated leg and leg segment occurred without noticeable changes to perfusion pressure or mean conduit artery diameter, while local tissue oxygenation, blood velocity, and blood flow were positively related to local temperature. Together, these findings support the notion that heat activates thermosensitive mechanisms in the leg microcirculation, thereby regulating the flow of blood through the human leg during local hyperthermia.

### 4.1 Impact of regional hyperthermia on leg tissue perfusion

Local hyperthermia, whether it be through prolonged whole or segmental leg heating, resulted in sustained ≥3‐fold increases in regional tissue perfusion. In all hyperthermic conditions, arterial blood flow was closely coupled with changes in local temperature—increasing exponentially with the rise in local hyperthermia. Of note is the strikingly similar increases in blood flow in the upper and lower leg during 1 h of segmental and whole leg hyperthermia (Figure 9). Previous studies have characterized the significant effects of segmental leg hyperthermia on global and local limb blood flow (Heinonen et al., 2011; Kuhlenhoelter et al., 2016; Romero et al., 2017; Thomas et al., 2017; Walsh et al., 2019), and the blood flow differences between the upper and lower leg under normothermia (Klein et al., 2003), and during whole body hyperthermia with lower leg occlusion (Chiesa et al., 2016). The present study is the first to comprehensively and directly compare the responses of the major leg arteries and the regional leg blood flow distribution during segmental and whole leg hyperthermia, in an attempt to isolate the effects of local thermosensitive regulatory mechanisms on thermal hyperemia. At baseline, we observed a ~ 3:1 distribution in blood flow between the upper and lower leg, in agreement with the previous literature (Chiesa et al., 2016; Klein et al., 2003). However, when upper and lower leg blood flow are expressed per 100 g of tissue, the blood flow values are similar, both at baseline and following 3 h of whole leg heating (2–3 and 9–10 ml min^−1^ 100 g^−1^, respectively). This suggests that the approximately three times greater mass (Wang et al., 1999) and, by extension, more abundant muscle, skin, fat, and bone vasculature of the upper leg in comparison to the lower leg, might largely account for the higher absolute blood flow of the upper leg. Notwithstanding, we consistently observed lower baseline tissue oxygenation in the lower leg and foot compared to the upper leg, suggestive of greater basal oxygen extraction from the circulation in response to lower local perfusion and tissue temperature (Davis et al., 2006). These indications of a coupling between lower temperature and blood perfusion in the lower leg and foot are consistent with observations that the distal regions of the leg are more susceptible to ischemia in disease conditions such as peripheral arterial disease and diabetes (Hills et al., 2009; Hirsch et al., 2006; Ouriel, 2001). Yet, in the present study, both leg segments were found to be highly responsive to prolonged leg hyperthermia, with the relative increase in blood flow being greater in the lower leg than the upper leg (3.8‐ vs. 2.6‐fold, respectively). Thus, the degree of tissue perfusion and temperature heterogeneity will, therefore, diminish with leg hyperthermia compared to control conditions (Figures 2 and 3). Nonetheless, the presently observed close temporal relationships between blood flow and local temperature and between regional tissue oxygenation and local temperature in both the experimental and control legs during the 6 h leg heating and recovery protocol, strongly support a causal link between local hyperthermia and hyperemia.

The blood flow responses of the major leg arteries to segmental leg heating reveal new insights into the regulation of tissue blood flow in human limbs during hyperthermia. Upper leg heating induced profound increases in thigh temperature and CFA blood flow (~3‐fold), yet lower leg temperature and POA blood flow decreased somewhat or remained unchanged (Figure 5). The observed upper leg hyperemia during the first hour of upper leg heating elicited similar increases in upper leg blood flow to those observed during whole leg hyperthermia (~370 ml min^−1^) and those reported in the literature during isolated limb heating and whole body heating (Chiesa et al., 2016; Heinonen et al., 2011; Pearson et al., 2011). In congruence with the findings during upper leg heating, lower leg and foot heating resulted in substantial increases in lower leg temperature and CFA, SFA, and POA blood flow, while PFA blood flow and upper leg temperature remained stable. The observation that the increase in CFA and SFA blood flow—which feeds into the POA—is similar to the increase in POA blood flow, lends additional support to the argument that upper leg blood flow remained unchanged. Furthermore, the magnitude of the lower leg hyperemia was similar to that evoked by 1 h of whole leg hyperemia (241–270 ml min^−1^; Figure 9). Moreover, increased tissue oxygenation—an index of microcirculatory blood flow—of the heated leg segment occurred in parallel to the regional hyperemia, while much like blood flow, no changes were observed in the non‐heated adjacent leg segment. Collectively, the similar magnitude of regional blood flow and tissue oxygenation, regardless of whether the heating intervention is being applied to a leg segment or the whole leg, provides additional compelling evidence supporting local hyperthermia as the putative stimulus for the greatly enhanced hyperemia.

### 4.2 Tissue perfusion regulation during local hyperthermia

Local hyperemia was associated with increases in local vascular conductance in all hyperthermic protocols, while perfusion pressure remained unchanged. A major aim of the present study was to identify the precise vascular locus in which hyperthermia modulates blood flow and tissue perfusion and in doing so, address the contribution between peripheral and central regulatory mechanisms. The primary forces that cause the movement of blood through a vessel are thought to be a positive pressure gradient and an increase in vascular conductance. Throughout all three heating protocols, no noticeable changes in perfusion pressure were observed—much like the majority of studies investigating the hemodynamic responses to local limb heating (Chiesa et al., 2016; Kalsi et al., 2017; Kuhlenhoelter et al., 2016; Pearson et al., 2011). In this study, segmental leg heating resulted in ≥3‐fold increases in tissue perfusion, and considerable increases in regional tissue oxygenation and skin blood flow, while the adjacent leg segment displayed no changes in these variables. Moreover, we observed no changes in cardiac stroke volume or wave intensity‐derived forward compression and forward expansion waves in the heated and control legs. This suggests that left ventricular contractility and late systolic flow deceleration were unchanged throughout the whole leg heating protocol even though cardiac output did increase to a similar extent as the rise in heated leg blood flow during the 3 h whole leg heating protocol (0.7–0.8 L min^−1^). Hence, the activation of regulatory mechanisms and pathways in the peripheral circulation rather than augmented central hemodynamic forces (Blair et al., 1960; Edholm et al., 1957; Roddie et al., 1957; Rowell, 1974) must explain the robust increases in blood velocity and flow in the vasculature of the limb hyperthermic tissues.

The increase in blood flow to the heated leg region occurred in the face of a maintained diameter in all examined conduit arteries, including those of the control leg and/or control leg segment where blood velocity and flow did not change. The present findings agree with studies reporting no changes in conduit artery diameter during limb heating (Chiesa et al., 2016; Coombs et al., 2019, 2021; Pearson et al., 2011; Teixeira et al., 2017), but are at odds with studies showing decreases in POA diameter (Thomas et al., 2017). The unchanged conduit artery diameter, together with the general increases in local vascular conductance with whole leg and segmental leg hyperthermia, suggest that vasodilatation might have instead occurred in the downstream small arteries and resistance arterioles, and/or alternatively thermosensitive physical and chemical mechanisms governing blood's rheological properties and kinetic energy permitted the increase in microvascular blood velocity and flow.

Interestingly, the studies assessing the impact of temperature variations in in vitro skeletal muscle vessel preparations show, for the most part, that the temperature *per se* does not exert a direct effect on smooth muscle contractile function (Ives et al., 2011; Vanhoutte & Shepherd, 1970). This lends support to the view that heat is predominantly acting indirectly via changes in temperature‐dependent blood viscosity, red blood cell deformability and dispersion, and/or intravascular vasodilatory mechanisms (Akyurekli et al., 1997; Artmann et al., 2008; Binzoni et al., 2012; Chiesa et al., ,^2015^, ^2016^; Heinonen et al., 2011; Stadler et al., 2012). Although not investigated herein, strong relationships exist between (1) increases in blood and tissue temperatures (Chiesa et al., 2015; Kalsi et al., 2017); (2) elevations in temperature and reductions in blood viscosity and frictional resistance (Çinar et al., 2001; Lim et al., 2010; Shin et al., 2004; Snyder, 1971); and (3) increases in temperature and rises in red cell deformability and dispersion (Çinar et al., 2001; Manteuffel‐Szoege, 1960, 1969; Pinho et al., 2016). Hence, heat *per se* may reduce blood viscosity and vascular resistance, and increase red cell deformability, red cell dispersion, blood velocity, and blood kinetic energy. It, therefore, seems plausible that heat‐modulated blood rheology explains at least part of the ≥3‐fold elevation in thermal hyperemia.

Another likely possibility is that local hyperthermia induces downstream vasodilatation via heat‐sensitive biochemical signals that (1) activate intravascular signaling‐transduction mechanisms in the microvasculature such as shear‐mediated nitric oxide release and cellular oxidative stress (Gifford et al., 2014; Kellogg et al., 1999; Laughlin et al., 2008; Minson et al., 2001; Paniagua et al., 2001; Romero et al., 2017) and/or (2) stimulate the release of vasoactive molecules from the circulating erythrocytes such as ATP (Kalsi et al., 2017; Kalsi & González‐Alonso, 2012; Pearson et al., 2011). In support of the involvement of red cell signaling mechanisms, close relationships have been reported between increases in temperature and erythrocyte‐derived ATP release, but not other blood constituents (Etulain et al., 2011; Kalsi et al., 2017; Kalsi & González‐Alonso, 2012), and between increases in plasma ATP and local limb hyperthermia (González‐Alonso et al., 2015; Kalsi et al., 2017; Kalsi & González‐Alonso, 2012; Pearson et al., 2011). Furthermore, increases in plasma ATP with intra‐arterial infusion cause profound elevations in limb blood velocity and tissue perfusion, independent of temperature, metabolic or perfusion pressure changes (González‐Alonso et al., 2008; Kalsi et al., 2017; Kalsi & González‐Alonso, 2012). Taken together, the present and previous experimental evidence suggest that local hyperthermia likely increases local perfusion through the activation of vascular thermosensitive mechanisms that cause microvessel vasodilatation.

### 4.3 Hyperthermia influence on local arterial distensibility

Another finding of the present study is that prolonged whole leg hyperthermia did not alter CFA distensibility in either the experimental or control legs. To our knowledge, this is the first study to directly assess the influence of limb hyperthermia on local arterial stiffness and distensibility using the ln(*D*)*U*‐loop method. Despite the ≥3‐fold increases in blood velocity, no changes were seen in wave speed or arterial distensibility. The present findings of local stiffness are in agreement with past whole body and two leg hyperthermia studies which reported no changes in regional (carotid–radial) arterial stiffness (Ganio et al., 2011; Moyen et al., 2016; Schlader et al., 2019). However, studies exploring the recovery following hyperthermia, reported decreases in peripheral and/or leg (femoral–ankle region) arterial stiffness alongside an elevated core temperature (Caldwell et al., 2017; Cheng et al., 2021; Lee et al., 2018; Sugawara & Tomoto, 2021; Thomas et al., 2017). The latter observations contrast with the unaffected or small changes in arterial stiffness/distensibility, core temperature, and arterial pressure observed in the present and previous single leg heating studies (Chiesa et al., 2015, 2016; Engelland et al., 2020; Takahashi et al., 2011). Previous studies have alluded to the possibility that a certain threshold of hyperthermic intensity—such that can initiate profound increases in core temperature and/or alterations in sympathetic activity—may be required to elicit reductions in arterial stiffness and associated increases in distensibility (Caldwell et al., 2017; Kaldur et al., 2016). Therefore, although further studies are warranted, the present findings indicate that single leg hyperthermia does not alter CFA stiffness and distensibility and thus, conduit artery vascular tone in conditions evoking no or negligible elevations in core temperature.

### 4.4 Perspectives and significance

The present study provides substantial evidence to indicate that limb tissue blood flow during regional hyperthermia is controlled by highly localized events in the microcirculation, as opposed to central hemodynamic forces or thermal reflexes responding to increases in core temperature. In this construct, the heart accommodates the increase in leg tissue perfusion by augmenting cardiac output, rather than playing a major role in the control of tissue blood flow and its distribution, as demonstrated here by (1) the unchanged PFA blood flow, quadriceps tissue oxygenation, and skin blood flow during lower leg and foot heating, despite the substantial increases in CFA, SFA, and POA blood flow (Figure 7), and (2) the unchanged POA blood flow and calf and foot tissue oxygenation during upper leg heating in the face of markedly elevated CFA blood flow (Figure 5). Future studies can use the approach employed by Watanabe et al., (2020) during exercise to directly test this hypothesis, by simultaneously measuring vascular and cardiac function during passive regional and systemic hyperthermia.

The profound and sustained increases in blood flow and shear rate with prolonged leg heating may serve as effective hemodynamic stimuli for improving vascular health. Shear stress—which increased ≥3‐fold in the present study—is widely accepted as an important stimulus for vascular remodeling (Girerd et al., 1996; Green et al., 2017; Vita et al., 2008; Zarins et al., 1987). Numerous intervention studies have investigated the effects of repeated two leg or whole body heating and the associated hyperemia on vascular health, reporting improvements in endothelial function (Brunt et al., 2016; Carter et al., 2014; Imamura et al., 2001; Kihara et al., 2002; Ohori et al., 2012). The advantage of the present local leg hyperthermia approach—in contrast to severe two leg or whole body heating interventions—is that the ensuing hemodynamic stimuli can be applied over prolonged periods without significant systemic physiological strain or thermal discomfort. Therefore, local limb heating may provide an effective alternative to promote beneficial arterial adaptations that improve vascular health in people with reduced or limited exercise capacity.

### 4.5 Experimental considerations

Some experimental considerations should be acknowledged when interpreting the present findings. As reported in the methods, different individuals participated in the three protocols which make this study a between‐subjects design as opposed to the gold standard within‐subjects design. Nevertheless, the data from the three participants completing all three protocols and published data (Chiesa et al., 2015, 2016; Keller et al., 2010; Pearson et al., 2011) during 1 h whole leg heating interventions in young healthy adults reveal comparable and reproducible leg blood flows to those observed in the present study (i.e., 0.5–0.6 ± 0.2 L min^−1^ increase in CFA blood flow). While the present protocols were different to address specific but complementary aims, the inter‐protocol comparisons were conducted during the first hour of each protocol when the methodology was identical, other than the obvious distinction of the heated region. Consequently, the highly reproducible hemodynamic responses to local leg and segmental hyperthermia and the fact that the responses were compared during the same timeframe, strongly support that our data and interpretations are valid and robust.

## 5 SUMMARY

In conclusion, the present study provides comprehensive and compelling evidence on the effects of local hyperthermia in human leg circulation. Prolonged whole leg hyperthermia produces a profound and sustained elevation in upper and lower leg blood flow, while segmental leg hyperthermia induces hyperemia to a magnitude that matches the regional hyperemia during whole leg heating without affecting blood flow, temperature or tissue oxygenation of the non‐heated limb segment. Increases in local tissue oxygenation, blood flow, vascular conductance, and blood velocity were positively related to the rise in local temperature, yet these increases occurred without any changes to mean perfusion pressure, conduit artery diameter or wave intensity‐derived parameters. Collectively, these findings support the notion that local hyperthermia increases peripheral tissue perfusion through the activation of local thermosensitive mechanisms. These mechanisms are proposed to increase microvascular blood flow by inducing blood rheology‐mediated increases in vascular conductance and/or vasodilatation in the microcirculation. The markedly enhanced hyperemia and tissue oxygenation strongly support the therapeutic potential of local hyperthermia for the treatment of circulatory diseases and/or rehabilitation.

## CONFLICTS OF INTERESTS

No conflicts of interest, financial or otherwise, are declared by the authors.

## AUTHOR CONTRIBUTIONS

This study was performed at Brunel University London, Uxbridge, UK. NKE and JGA conceived and designed the research. NKE, ORG, AWK, and JGA acquired the data. NKE analyzed the data. NKE, AWK, and JGA interpreted the data. All authors revised the manuscript and provided intellectual feedback and agreed to be accountable for all aspects of the work.

## Data Availability

The raw, unidentified data collected throughout this study is available via Brunel Figshare, an online data repository database. https://doi.org/10.17633/rd.brunel.14749386.
